# The Female Menstrual Cycles Effect on Strength and Power Parameters in High-Level Female Team Athletes

**DOI:** 10.3389/fphys.2021.600668

**Published:** 2021-02-22

**Authors:** Marcus S. Dasa, Morten Kristoffersen, Elisabeth Ersvær, Lars Peder Bovim, Lise Bjørkhaug, Rolf Moe-Nilssen, Jørn V. Sagen, Inger Haukenes

**Affiliations:** ^1^Department of Global Public Health and Primary Care, University of Bergen, Bergen, Norway; ^2^Department of Sport, Food, and Natural Sciences, Western Norway University of Applied Sciences, Bergen, Norway; ^3^Department of Safety, Chemistry, and Biomedical Laboratory Sciences, Western Norway University of Applied Sciences, Bergen, Norway; ^4^Department of Health and Functioning, Western Norway University of Applied Sciences, Bergen, Norway; ^5^Department of Clinical Science, University of Bergen, Bergen, Norway; ^6^Department of Medical Biochemistry and Pharmacology, Haukeland University Hospital, Bergen, Norway; ^7^Research Unit for General Practice, NORCE Norwegian Research Centre, Bergen, Norway

**Keywords:** menstrual cycle, female athletes, strength, power, hormones

## Abstract

**Purpose:**

The female menstrual cycle (MC) is characterized by hormonal fluctuations throughout its different phases. However, research regarding its effect on athletic performance in high level athletes is sparse. The aim of this study was to (i) investigate the female MCs effect on strength and power performance in highly trained female team athletes throughout the MC and (ii) examine whether eumenorrheic participants with natural hormonal fluctuations displayed enhanced performance in the follicular phase (FP) versus the luteal phase (LP), compared to controls using hormonal contraceptives.

**Materials and Methods:**

A total of 29 athletes (Age 21.2 ± 3.3 years; weight 65.6 ± 8.7 kg; height 170.2 ± 8.0 cm; and fat free mass 52.7 ± 7.1) completed the study after a 6-week testing period (8 eumenorrheic participants and 21 hormonal contraceptive controls). Participants were recruited from the team sports soccer, handball and volleyball. Testing protocol consisted of maximal voluntary isometric grip strength, 20-m sprint, countermovement jump and pneumatic leg-press. Based on self-reported use of hormonal contraceptives, participants were divided into non-hormonal contraceptive group and hormonal contraceptive group, the latter working as a control group. Differences in performance between the FP and LP were investigated. MC phase was confirmed by serum hormonal levels through venous blood samples in the non-hormonal contraceptive group.

**Results:**

There were no statistically significant changes for the two different phases of the MC, in terms of physical performance for the whole group. Further, there was no significant difference between groups during the MC for any of the outcome variables, maximal voluntary isometric grip strength *F*(3.29) = 0.362; 20-m sprint *F*(3.24) = 0.710; countermovement jump *F*(3.26) = 2.361; and leg-press *F*(3.26) = 1.746.

**Conclusion:**

In high level female team athletes, no difference in performance was observed based on hormonal contraceptive status. This suggests that the MC does not alter acute strength and power performance on a group level in high level team athletes.

## Introduction

Modern intermittent team sports require a wide range of physiological attributes in order to meet the demands of the given sport. Strength and power are important characteristics in intermittent team sports, hence, compelling attributes to research in relation to physical performance (Drust et al., 2007; Lidor and Ziv, 2010; Póvoas et al., 2014). Although gender equality has been more in focus in recent years, related to exposure, conditions and professionalism in sport, females are underrepresented in the scientific literature. Findings from studies investigating male physiology and performance does not necessarily translate to females, as the female menstrual cycle (MC) cause monthly changes in serum hormone levels (Reilly, 2000) that may affect strength, power, and endurance (Hansen, 2018; Thompson et al., 2020).

The role of the sex hormones (estradiol and progesterone) in addition to the pituitary hormones regulating the gonadal axis [i.e., lutenizing hormone (LH)- and follicle stimulating hormone (FSH)], in developing strength and power, is not fully understood. Studies suggest that estradiol, one of the primary sex hormone during the reproductive MC, induce anabolic, and muscle building processes in females (Lowe et al., 2010; Hansen et al., 2012). Furthermore, hormone replacement therapy using exogenous estrogen seem to attenuate loss of muscle strength in peri and post-menopausal women (Carter et al., 2001; Minahan et al., 2015). The impact of estradiol on muscle strength is not necessarily accomplished by increased muscle size through hypertrophy, but rather by affecting the intrinsic quality of skeletal muscle, enabling muscle fibers to generate greater force (Lowe et al., 2010). However, the role of estradiol during acute hormonal changes, as seen amid the MC is not fully understood (Chidi-Ogbolu and Baar, 2019). A second hormone playing an important role during the female MC is progesterone. A decline in progesterone levels (and estradiol levels) signals shedding of the endometrium resulting in menstrual bleeding. Related to sports, progesterone has been associated with protein catabolism, conceivably attenuating muscle strength (Oosthuyse and Bosch, 2010). Indeed, studies have demonstrated ameliorated strength levels during both the follicular phase (FP) and luteal phase (LP) (Phillips et al., 1996; Birch and Reilly, 2002). These conflicting finding makes understanding of potential superior performance allocated to a specific MC phase speculative. Sung et al. (2014) demonstrated increased muscle strength and muscle diameter through strength training periodized to the FP, compared to LP (Sung et al., 2014). Wikström-Frisén et al. (2017) confirmed this finding and reported increased power and strength gains through FP periodized training (Wikström-Frisén et al., 2017). Despite this, several studies within this field of research are hampered with insufficient methodology, by lacking biological documentation of the MC phase, which is seen as the gold standard (Allen et al., 2016; Janse et al., 2019; Thompson et al., 2020). Studies with unsatisfactory biological methodology also report increased muscular performance through changes in serum hormonal levels during and shortly prior to menstrual bleeding (Sarwar et al., 1996; Bambaeichi et al., 2004).

A recently published systematic review investigating the effect of MC and oral contraceptives on acute responses and chronic adaptations to resistance training, concluded that the effects of both the MC and oral contraceptive use on acute responses to resistance training remain unclear (Thompson et al., 2020). To summarize some findings of this review: (i) One of the 17 studies found no significant differences in acute responses to a resistance training session over the natural MC, while four studies did find changes (Thompson et al., 2020). (ii) For the responses to a resistance training program, three studies reported FP -based training to be superior to LP -based training or regular training, while one study reported no differences (Thompson et al., 2020). Finally, (iii) when assessing the differences in acute responses between the oral contraceptive and MC groups, two studies reported oral contraceptives to have a positive influence, whilst four studies reported that oral contraceptive users had a delayed recovery, higher levels of markers of muscle damage, or both (Thompson et al., 2020). Some of the conclusions made by this review is that FP -based resistance training programs appear to result in better responses than LP based and regular training programs. Furthermore, this review highlights the need for further experimental research in this area (Thompson et al., 2020).

We argue that more research is needed to investigate whether hormonal fluctuations during the MC may alter performance and in such have implications for periodic training and competition. Furthermore, no studies have investigated the effect of female sex hormone fluctuations on performance in high-level team athletes, a sub-group where small changes may have major impact. The aim of the study was therefore (i) to examine potential changes in performance in high level female team athletes during different phases of the MC and (ii) to examine whether eumenorrheic participants, with natural hormonal fluctuations during the MC display enhanced performance during the FP versus the LP (due to higher relative concentrations of circulating estradiol), compared to participants using hormonal contraceptives.

## Materials and Methods

### Participants

Women from six high-level teams from the Hordaland County of western Norway, Norway, were invited to participate in the study. Team sports invited were soccer, handball and volleyball, competing at the top two national levels. Twelve players were currently representing their national team. Five teams accepted the invitation; one team declined due to logistics. Inclusion criteria were (i) ≥18 years of age, (ii) competing at a national level in their respective team sport, and (iii) free of any injury or disease prohibiting testing. All participants satisfying inclusion criteria (*n* = 55) received a baseline questionnaire via email before the start of the intervention period. Of these, nine team-members dropped out before testing, leaving the final participant number at 46.

**FIGURE 1:**
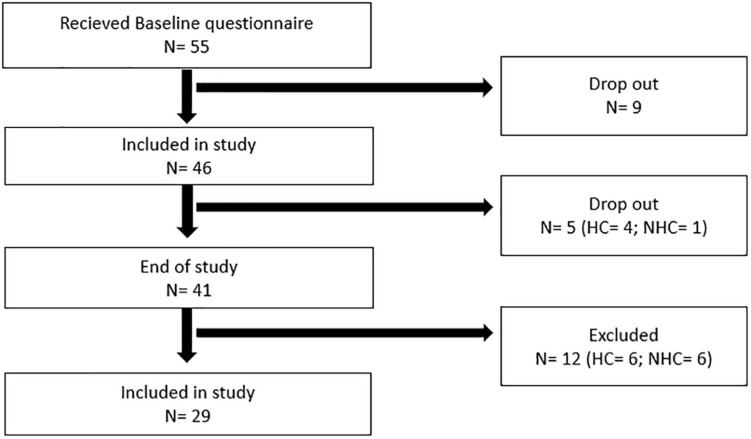
Flow chart displaying the drop-out rate and exclusion during the study. N, number of participants; HC, hormonal contraceptive user; NHC, non-hormonal contraceptive user. HC status for drop out prior to the start of the study is not included, due to incomplete questionnaires from some of the participants.

During the testing period, additional five participants were lost to follow up due to time commitment, school, or injury. Four participants were excluded *post hoc* as they did not self-report onset of menstruation during the 6-week testing period. Additionally, eight participants were excluded as they did not provide at least two test weeks from both FP and LP. Thus, the total number of participants completing the study was Figure 1 (Age 21.2 ± 3.3 years; weight 65.6 ± 8.7 kg; height 170.2 ± 8.0 cm; and fat free mass 52.7 ± 7.1). Group specific descriptive data are presented in the section “Results.”

This study was approved by the Regional Ethics Committee Norway (REK2018/1529).

### Study Design

A prospective cohort study with repeated measures over 6-weeks was executed. Exposure was hormonal contraceptive (HC) status. Hormonal contraceptive group (HCG) and non-hormonal contraceptive group (NHCG) were defined *post hoc* (the participants HC status was unknown during the 6-week testing period). The MC phases (FP and LP) were confirmed through serum hormonal levels in blood samples taken at every visit during the 6-week testing period, in the NHCG, to ensure correct group assignment and validity of self-reported menstrual phase. This validation procedure (hormonal values in serum samples) was not performed in the HCG, as confirmation of MC phase through blood samples is not possible due to a steady flow of contraceptive specific exogenous sex hormones throughout the MC (Allen et al., 2016). Self-reported onset of menstruation was used in this group for assignment of FP and LP phases. Guidelines provided by Sims and Heather (2018) were used to choose test weeks included in the statistical analysis for the HCG (Sims and Heather, 2018). More specifically, we strived to test participants in the HCG using monophasic oral contraceptives in the active pill phase. Blood samples of subjects in the HCG were, however, drawn, but for other analytical purposes (parallel study). HC types were recorded at baseline in the HCG (Supplementary Table 3). Weekly outcome measures (see testing procedure) were compared between the HCG and NHCG. For this, four out of six testing weeks were selected for statistical analysis to represent the FP and LP phases (FP week 1/2 and LP week 3/4). Six weeks of consecutive testing was performed to secure a minimum of two measurements in both FP and LP, thus minimizing exclusion based on failure to fulfill this requirement (e.g., week 1-4 and week 5-2 etc.). This variation of included test weeks reduced the chance of a systematic learning effect.

### Testing Procedures

At baseline, all participants completed the LEAF-Q questionnaire: a screening tool for the identification of female athletes at risk for the female athlete triad. The questionnaire comprises questions of age, height, weight, and BMI, as well as more comprehensive information (sleep, nutrition, and metabolism) for purposes beyond the scope of this study (Melin et al., 2014).

On the day of testing, participants were encouraged to eat approximately the same type and amount of food and liquid throughout the testing period. They were also instructed to avoid any caffeine consumption 12 h preceding testing, due to the possible ergogenic effects related to physical performance (Goldstein et al., 2010; Grgic et al., 2019a). When arriving at the facility for testing, participants were asked to complete a self-administered questionnaire regarding MC, nutrition, training, injuries/pain, and test-day form (expectations on test influence). Weight and body composition were measured using multi-frequency bioelectrical impedance (IN-body 720, Biospace, Tokyo, Japan), along with venous blood samples for determination of menstrual phase. Before physical testing, participants performed a 15-min standardized warm up (100 watts) using the Wattbike ergometer (Wattbike Ltd., Nottingham, United Kingdom). The weekly testing protocol consisted of maximal voluntary isometric grip strength (MVIGS), 20-m sprint, Counter movement jump (CMJ) and leg-press. Testing was completed once a week, at approximately the same time each weekday to control for circadian variations (±2 days/±2 h on the testing day). To minimize potential bias through a learning effect, participants were given two experimental attempts for every test, before recording started.

### Maximal Voluntary Isometric Grip Strength (MVIGS)

Isometric grip strength of the dominant hand was measured using a digital pinch/grip analyzer (MIE, Medical research Ltd, Leeds, United Kingdom). Standard instructions before and during the test: (i) to be seated with a slight forward bend of the trunk, elbow resting on the thigh with 90° elbow flexion, (ii) exert maximal force for 3–5 s, (iii) two attempts with a 30 s break between. If deviation from the instructions the attempt was disallowed. If the force production in their last attempt exceeded the previous with >5%, a new attempt was performed. The best recording was used for statistical analysis. MVIGS is shown to be a repetitive measure, with ICC-scores classified as very good (Vassilis, 2012).

### 20-M Sprint

Sprint performance was assessed over a 20-m track, made from portable non-slipping surface (Hitashita international, ON, Canada). Times were recorded at 5, 10, and 20-m using single beam photocells (Brower timing systems, Utah, United States). Photocells were fixed at 120 cm height, apart from the first pair, which were fixed at 10 cm height to ensure similar photocell interception. participants were instructed to a stationary start with the dominant leg behind and a slight forward bend of the trunk. Participants decided when to start their effort, with recording being initiated by interception of the first photocell beam. Each subject carried out two attempts, separated by 2 min of rest. If the second attempt was > 5% faster than the previous, a new attempt was performed. The best total 20-m sprint time was used for statistical analysis. 20-m sprint is shown to be a repetitive measurement, with ICC-scores classified as very good (Shalfawi et al., 2012). This method is also reviewed as reliable in a comprehensive review of sprint performance monitoring (Haugen and Buchheit, 2016).

### Countermovement Jump

Counter movement jump performance started from an upright position and the participants were instructed to descend to a self-chosen depth, followed by a maximal vertical jump effort. Participants were instructed to use a hand on hips position throughout the entire movement. Maximum jump height (cm) was calculated using Kistler Measurement, Analysis and Reporting Software (MARS, 2015, S2P, Ljubljana, Slovenia). Participants performed at least two attempts or continued until performance declined. The best attempt was selected for statistical analysis. CMJ is shown to be a repetitive measure, with ICC-scores classified as very good (Slinde et al., 2008).

### Leg Press

Relative peak power (RPP) was estimated using a progressive maximal pneumatic resistance seated leg press test on a Keiser A420 (Keiser, Fresno, CA, United States). An estimated 1-Repetition maximum (1 RM) was chosen based on participants self-reported training history, bodyweight, sport and previous internal testing. Participants completed a 10-repetition incremental step test toward peak of 1 RM on last repetition. The test protocol is well established and described in detail elsewhere (Redden, 2019). RPP was estimated using all 10 repetitions, and each participant was to use the same protocol at every test point. RPP measured and estimated with pneumatic leg press is shown to be a repetitive measure, with ICC-scores classified as good (Redden et al., 2018).

### Serum Hormone Analysis

To provide hormonal confirmation of MC phase, participants provided non-fasted venous blood samples before testing at every visit, in order to avoid effect of physical strain on hormonal levels. Blood was collected using eclipse blood collection needles (BD vacutainer, Franklin Lakes, NJ, United States) in EDTA-vials by qualified biomedical laboratory scientists. After 30 min, blood samples were centrifuged at 2000×*g* for 10 min (Thermo Fisher Scientific SL1R centrifuge, Thermo Fisher Scientific, Waltham, MA, United States). Serum was isolated and stored at −80°C until all samples were collected during the 6-week testing period prior to hormonal analysis. Serum samples were analyzed for progesterone, estradiol, FSH, LH, and Sex hormone-binding globin (SHBG). Analysis of progesterone and estradiol levels were performed using Liquid Chromatography-Mass Spectrometry (LC-MS/MS) (progesterone by the LC-system by Agilent (Santa Clara, CA, United States) and MS-system: SICX, while estradiol by LC-MS/MS by Waters). FSH, LH and SHBG was analyzed by chemical luminescence on the Immulite 2000 XPi (Siemens, Erlangen, Germany). All hormone analyses were performed at the Hormone Laboratory, Department of medical biochemistry and pharmacology, Haukeland University Hospital. All analyses are accredited according to NS-EN ISO 15189:2012. Reference values used by the laboratory are presented in Supplementary Table 4.

We performed a dichotomous division for FP and LP, respectively. A progesterone value < 0.5 nmol/L was considered true FP whereas a value > 5.5 nmol/L was considered true LP. In some instances, we identified progesterone levels between 0.5 and 5.5 nmol/L. For these cases we evaluated the preceding and the following weeks progesterone values. If the week prior presented a progesterone value of >5.5 nmol/L, both weeks were considered to be LP. In other cases, a low but measurable progesterone value appeared, followed by a spike in progesterone. These cases were labelled mid-cycle and were not included in the statistical analysis, since it is difficult to conclude whether the value is actually late FP or early LP.

### Statistical Analysis

Descriptive statistics were used to examine the distribution of subject characteristics across exposure groups. We calculated interclass correlation coefficients (ICC, random model) as measures of reliability, based upon the four weekly measurements chosen for analysis of performance during the MC. The results are presented as mean ± standard deviation (SD). One-way repeated measures analysis of variance (ANOVA) were used to analyze for between group differences, between the average results of two FP measures and two LP measures. ANOVA was also conducted separately for the HCG and NHCG to investigate potential within group chances between the FP and LP. Bonferroni correction was conducted *post hoc* to reduce the chance of type one error with significant ANOVA’s. An alpha level of *P* < 0.05 was set *a priori*. Participants with incomplete hormonal or performance data were excluded from the statistical analysis. Between groups comparison is based on the interaction between hormonal status^∗^menstrual cycle (time). Statistical analysis was performed using IBM SPSS 25 (IBM, Armonk, NY, United States).

## Results

A total of 29 participants were included in the study, 21 and 8 individuals in the HCG and NHCG, respectively. Mean age, height, weight, fat free mass, and monthly training volume are presented in Table 1. There was no statistical difference between the two groups for any of these measures.

**TABLE 1:** Descriptive data showing mean values ± standard deviation of subject characteristics in the hormonal contraceptive group and non-hormonal contraceptive group.

	Total	HCG	NHCG
*N*	29	21	8
Age	21.2 ± 3.3	20.48 ± 2.5	22.5 ± 4.2
Weight (kg)	65.6 ± 8.7	66.9 ± 8.3	63.1 ± 9.3
Height (cm)	170.2 ± 8.0	170 ± 7.6	168.8 ± 8.9
Fat free mass (FFM)	52.7 ± 7.1	53.4 ± 6.7	52.3 ± 7.9
Training volume (monthly hours)	52.8 ± 14.2	52.7 ± 14.9	52.9 ± 13.7

*N, number of participants.*

Self-reported onset of menstruation was registered in the NHCG (*n* = 8) and compared with hormonal analyses to investigate the accuracy of self-reported onset of menstruation. We found deviating results in the self-reported data in two individual cases (ID: D16, H3). Range of analyses in the different phases of MC are presented in Supplementary Table 1. Reference values for FP and LP are presented in Supplementary Table 4.

Repeated measures reliability was assessed as satisfactory to excellent, with the following ICC vales; 0.75 for countermovement jump, 0.80 for leg press, 0.88 for MVIGS, and 0.91 for 20-m sprint performance.

**FIGURE 2:**
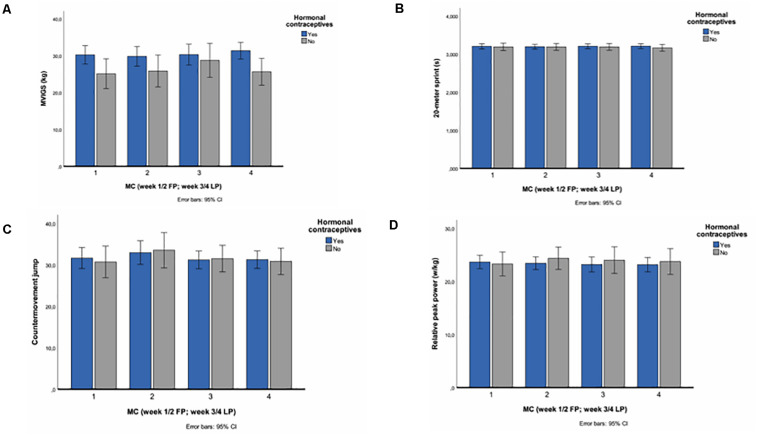
Mean values through the menstrual cycle with 95% confidence intervals for **(A)** maximal voluntary grip strength, **(B)** 20-m sprint, **(C)** leg press, and **(D)** countermovement jump. Week 1–4 (week 1/2 FP; week 3/4 LP) are based on serum hormonal levels.

There were no statistically significant changes for the two different phases of the MC, in terms of physical performance for the whole group. Further, the interaction effect between contraceptive status and time (MC phase) were compared. There were no statistically significant group differences between the HCG and NHCG for any outcome measures throughout the 4 weeks chosen for analysis, although both groups showed variation in performance (Figure 2 and Supplementary Table 2). Additionally, no significant changes were observed when investigating the MC cycle in both groups separately.

Despite non-significant findings, In terms of *maximal isometric grip strength* both groups demonstrated weekly variations in MVIGS, displaying the highest isometric grip strength values for both groups in the LP with a mean of 31.3 ± 5.6 kg in the HCG and 28.7 ± 3.6 in the NHCG, respectively (Figure 2 and Supplementary Table 2). Further, both groups showed consistent measures for *sprint performance* throughout the 4 weeks of the MC, with results ranging from 3.194 ± 0.132 s to 3.209 ± 0.123 s in the HCG and 3.165 ± 0.121 s to 3.191 ± 0.116 s in the NHCG, respectively (Figure 2 and Supplementary Table 2). With respect to *counter movement jump* the CMJ displayed a greater between group difference compared to the other tests, with means ranging from 32.9 ± 6.3 cm to 30.7 ± 4.3 cm in the HCG and 33.5 ± 4.7 cm to 30.7 ± 2.7 cm in the NHCG, respectively (Figure 2 and Supplementary Table 2). Finally, the NHCG displayed greater variation between weeks in *leg press* compared to the HCG; means ranging from 24.4 ± 2.9 W/kg to 23.3 ± 2.0 W/kg in the NHCG compared to 23.6 ± 2.6 W/kg to 23.2 ± 2.8 W/kg in the HCG (Figure 2 and Supplementary Table 2).

## Discussion

The aim of this study was to examine potential changes in performance due to hormonal changes during the MC. This was done by comparing test results from FP and LP between a NHCG and HCG, the latter serving as controls over a 6-week period. When comparing eumenorrheic participants with controls using HC, we found no statistically significant differences for the MC overall. Further, no statistically significant differences were found for the FP when comparing the two groups. These findings suggest that HC status does not alter strength and power performance on a group level in high-level female team athletes, when tested in a controlled environment throughout one MC.

Our findings are in line with several studies reporting no difference in strength or power performance throughout the different phases of the MC (Lebrun et al., 1995; Jonge et al., 2001; Fridén et al., 2003; Bushman et al., 2006; Abt et al., 2007; Julian et al., 2017; Romero-Moraleda et al., 2019). However, there are studies reporting altering effects on strength and power performance following periodized training regiments, adjusting for hormonal fluctuations in the different MC phases (Thompson et al., 2020). For instance, Sung et al. (2014) reported increased muscle strength in participants following a FP periodized training regimen, compared to a LP periodized training regiment, over a total of three MC’s (Sung et al., 2014). Possibly, this procedure, in contrast to acute performance as investigated in our study, could better utilize the proposed mechanisms behind estradiol’s ameliorating effects in muscle strength (Lowe et al., 2010). Our study was cross-sectional, and we did not follow any specific strength intervention and only provided data for one MC. This might explain why the NHCG did not display any significant alterations in their performance relative to the HCG. Further, Phillips et al. (1996) reported up to 10% increase in maximal voluntary force of the adductor pollicis during the FP of the MC (Phillips et al., 1996). These findings contradict our result, although we used MVIGS with a handheld dynamometer, which is comparable with maximal voluntary force of the adductor pollicis. Although not significant, in the current study, MVIGS demonstrated small weekly variations in performance for both groups with the greatest variations between FP and LP for both groups. As the weeks are based on self-reported menstruation and confirmation with hormonal analyses, representing the biological week of the MC and not chronological test week, a systematic learning effect can be ruled out. The fact that both groups showed similar variations in their results, suggests that the MC was not the impacting factor in the NHCG.

Counter movement jump displayed slight weekly non-significant variations in the NHCG throughout the MC. In contrast, the HCG showed a substantial deviation with a mean difference of 2.2 cm between week two and four, which is a 6.9% difference. This manifested itself with a FP peak before declining in the LP and can be viewed as a meaningful difference, considering the marginal differences in high level sport. If estradiol indeed does ameliorate muscle strength and power, this increase in performance would be plausible. However, with HC agents providing a steady supply of exogenous hormones throughout the MC, minimizing the hormonal fluctuations, this weekly difference in the HCG was unexpected. As highlighted by Myllyaho et al. (2018), different HC agents may exert varying effects of performance (Myllyaho et al., 2018). Hence, this might be a line of investigation interesting to follow in future studies, by separating groups of different HC agents.

Relative peak power showed weekly variations, with the NHCG displaying the greatest change between week one and two, with a mean difference of 1.1 w/kg. For this outcome, the greatest value manifested itself in the FP. This is in line with previous research, demonstrating superior performance in the FP (Phillips et al., 1996; Greeves et al., 1997). However, the outcome measures of both these studies are quite reductionistic. Further, MC phase was not necessarily verified through serum blood samples, giving these results low validity (Allen et al., 2016).

A total of 20-m sprint displayed the least variation in both groups. Since repeated measures ICC was high for sprint measures, these results cannot be explained as uncertainty of the measurement method to detect small changes, but rather demonstrates stable performance across the testing period. Further, it is possible 20-m was too short of a distance to measure any meaningful difference, as results were very marginal.

Although no significant findings were detected between groups, several variables showed substantial weekly variations in performance in both the HCG and NHCG. As small changes in performance may impact results in high level sport, our findings indicate that the time of testing could yield different results for high level female athletes in general. However, these variations cannot directly be contributed to hormonal fluctuations occurring during the MC. One plausible explanation for the weekly variations observed in this study is individual physiological load. Indeed, Andersson et al. (2008) demonstrated that full match recovery in female soccer players can last up to 72 h. The recovery time from handball is also proven to be substantial (Ronglan et al., 2006; Andersson et al., 2008).

Our study contradicts the suggestion that estradiol acutely ameliorates muscle strength and power, supporting the findings of Jonge et al. (2001), showing no correlation between estradiol serum hormonal levels and muscle strength, fatiguability, and contractile properties (Jonge et al., 2001). Serum estradiol levels varied in the range from 35 pmol/L to 1122 pmol/L. As highlighted by Jonge et al. (2001), the large variations seen in serum estradiol levels is partly caused by secretory pulses of these hormones. In addition, the MC is highly individual in their length, thus, the participants may have been in different stages of their respective cycles. This is in line with Greeves et al. (1997) who demonstrated that supraphysiological doses of estradiol did not significantly increase muscle strength, questioning the findings of Phillips et al. (1996), and Sarwar et al. (1996), suggesting that acute changes in estradiol ameliorates muscular strength (Phillips et al., 1996; Sarwar et al., 1996).

Indeed, several studies have shown estradiol hormone replacement therapy to be beneficial in decreasing attenuation of muscle strength in peri and post-menopausal women, as summarized by Chidi-Ogbolu & Baar (Chidi-Ogbolu and Baar, 2019). Lowe et al. (2010) hypothesize that estradiol alters myosin function during muscle contractions through estradiol receptors in a typical steroid manner (Lowe et al., 2010). As described by the aforementioned authors, skeletal muscle is an estradiol receptive tissue, working through the estradiol receptor. This may help explain why studies adopting periodized training protocols, utilizing elevated circulating estradiol levels over time have demonstrated increased muscle strength, as opposed to more modest results displayed when measuring acute performance. Exploiting a preferable milieu may promote strength gains over time, as adaptations in skeletal muscle may need time to manifest themselves, even if estradiol alone also improves the intrinsic quality of contractile fibers. Individual estrogen receptor content may therefore be partly indicative of muscle strength gains and performance.

As highlighted by Sims and Heather (2018), HC users are proposed as suitable controls when measuring the effects of the MC (Sims and Heather, 2018). However, several types of contraceptive agents, with different active ingredients and exogenous hormones were administered in the HCG. Thus, the impact on performance may vary, depending on the type of contraceptive agent being used. To our knowledge, there is no study investigating the effect on different type of HC’s related to athletic performance in high level athletes. Considering the current lack of knowledge related to different HC agents and performance, we would like to re-emphasize the need for future research regarding this manner.

### Methodological Considerations and Limitations

This study highlights the possibility of conducting prospective research on female athletes, which is often considered problematic due to training and competition schedule, paired with female physiology. A further strength is the prospective study design, were we measured 6 weeks and chose 4 weeks, representing the MC based on self-reported onset of menstruation and hormonal confirmation. However, measurements over several MC’s would have provided increased validity, due to individual variation in MC length and onset. Comparing self-reported onset of menstruation to hormonal analysis, revealed deviating results in four participants. Although some of these variations may be contributed to survey misunderstandings, the results indicate that some caution should be made when interpreting studies exclusively applying this method for MC phase determination. Although no statistical difference was observed between the two groups, some interindividual variability was present in both groups. With a relatively small sample size, it is possible that the results would approach being statistically significant with a larger population. Further, a potential limitation is small variations in test procedures. Testing was originally scheduled for the same day (± 2 days) and time (±2 h) throughout the study period to account for circadian variations. However, due to national team obligations and training/competition schedules being fluctuant in pre-season, some participants were tested outside these frames at one or several time points. This could have impacted the results, as circadian rhythm has been shown to affect performance in strength and power endeavors (Grgic et al., 2019b). Grgic et al. (2019b) reported that expression of strength is greater in the evening, versus the morning. On the other hand, the circadian effects on test results also depend on athletes normal training schedule (Grgic et al., 2019b). Thus, the impact of variation in test procedures on the final results is not fully known. Indeed, the relatively low sample size limits external validity. On the other hand, the participants were selected based on stringent criteria’s before entering the study, increasing the likelihood of being representative for high level female team athletes within the relevant sports. As described earlier, HCG testing weeks was selected based on self-reported menstruation. Although we strived to follow guidelines presented by Sims and Heather (2018), this method of validation should be interpreted with caution (Sims and Heather, 2018). Finally, we chose to limit testing to FP and LP exclusively. Ideally, hormonal validation of MC phase should have included ovulation. Further, FP and LP could have been divided to include early and late FP and LP. Due to time and financial reasons, this was not done. Hence, this limits our result, due to variations in serum hormonal levels in different parts of FP and LP, respectively.

## Conclusion

In this study, there was no statistically significant changes for the two different phases of the MC, in terms of physical performance in high level female team athletes. Further, testing of strength and power performance parameters were not significantly affected by HC status when comparing an HCG and NHCG during the MC. These findings suggest that MC phase should not be of major consideration for athletic testing or competition, emphasizing strength and power performance. However, interindividual variability in results and hormonal levels, together with a small sample size makes firm guidelines arduous. Based on our study and the current literature, possible alterations to strength and power performance elicited by the MC are more likely to occur during long term MC phase dependent training interventions, rather than acute testing of performance. Our findings should be interpreted with caution, due to the limitations of the study.

## Data Availability Statement

The raw data supporting the conclusions of this article will be made available by the authors, without undue reservation.

## Ethics Statement

The studies involving human participants were reviewed and approved by Regional Committees for medical and health research ethics (2018/1529). The patients/participants provided their written informed consent to participate in this study.

## Author Contributions

MD: planning, data collection, statistical analysis, and writing. MK and LPB: planning and data collection. EE: planning and writing. LB: planning, data collection, and writing. RM-N: statistical analysis. JS: writing and hormonal data analysis. IH: planning, statistical analysis, and writing. All authors contributed to the article and approved the submitted version.

## Conflict of Interest

The authors declare that the research was conducted in the absence of any commercial or financial relationships that could be construed as a potential conflict of interest.
